# Sepsis recognition in the emergency department – impact on quality of care and outcome?

**DOI:** 10.1186/s12873-017-0122-9

**Published:** 2017-03-23

**Authors:** Marius Morr, Alexander Lukasz, Eva Rübig, Hermann Pavenstädt, Philipp Kümpers

**Affiliations:** Department of Medicine D, Division of General Internal Medicine, Nephrology, and Rheumatology, University Hospital Münster, Albert-Schweitzer-Strasse 33, 48149 Münster, Germany

**Keywords:** Sepsis, Severe sepsis, Sepsis recognition, Infection, Emergency department, Quality of care

## Abstract

**Background:**

Appropriate and timely recognition of sepsis is a prerequisite for starting goal-directed therapy bundles. We analyzed the appropriateness of sepsis recognition and documentation with regard to adequacy of therapy and outcome in an internal medicine emergency department (ED).

**Methods:**

This study included 487 consecutive patients ≥18 years of age who presented to a university hospital ED during a 4-week period. Clinical, laboratory, and follow-up data were acquired independently from documentation by ED physicians. The study team independently rated quality of sepsis classification (American College of Chest Physicians/Society of Critical Care Medicine definitions), diagnostic workup, and guideline-adherent therapy in the ED.

**Results:**

Of 487 included patients, 110 presented because of infection. Of those, 54 patients matched sepsis criteria, including 20 with organ damage and thus severe sepsis, as rated by the study team. Sepsis was not recognized in 32 of these 54 cases (59%). Multivariate binary logistic regression analysis revealed that higher systolic blood pressure (*p* <0.05), the ability to stand (*p* <0.01) and a low number of documented vital signs in the ED discharge letter (*p* < 0.05) were independent predictors of missed sepsis. Surprisingly, adequate detection of the septic focus (81 vs. 93%, *p* = 0.17), appropriate fluid administration (86 vs. 87%, *p* = 0.39), and guideline-adherent antibiotic regimen (95 vs. 100%, *p* = 0.42) did not differ between cases of recognized and unrecognized sepsis, respectively. Non-recognition affected neither death-censored length of hospital stay (median 7.63 d vs. 7.13 d, *p* = 0.42) nor a combined endpoint of death or ICU admission to (9 vs. 12%, *p* = 0.55).

**Conclusions:**

Non-recognition of sepsis in ED patients with serious infections who formally meet organizational sepsis definitions seems to have no deleterious impact on initial therapy adequacy.

**Electronic supplementary material:**

The online version of this article (doi:10.1186/s12873-017-0122-9) contains supplementary material, which is available to authorized users.

## Background

Sepsis is a leading cause of death in intensive care units (ICUs) of western countries, associated with enormous healthcare costs and long-term morbidity [1]. Based on a large prospective study, the German competence network “SepNet” extrapolated an annual incidence of 79,000 sepsis cases and 75,000 severe sepsis cases in German ICUs, accounting for almost 60,000 deaths [2]. These data clearly do not agree with the official number of cases compiled via International Statistical Classification of Diseases (ICD) classification (39,000 cases; 6,000 deaths), indicating that sepsis may be under-recognized in clinical routine [3]. Given that approximately every third patient with severe sepsis is admitted through the emergency department (ED) [4, 5], recognizing these cases and initiating appropriate treatment is of utmost importance. Unfortunately, sepsis recognition is hampered by complex and changing definitions. Originally introduced in 1992 [6], a sepsis diagnosis was based on at least two of the following systemic inflammatory response syndrome (SIRS) criteria attributed to a presumed infection: (1) body temperature >38 °C or <36°; (2) heart rate >90 beats per minute; (3) respiratory rate >20 breaths per minute; and (4) white blood cell count >12,000/mm^3^ or <4,000/mm^3^. Over the years, the concept of SIRS has been increasingly criticized for limited specificity and sensitivity [7–10] and was replaced in 2001 with a longer list of potential signs and variables [11]. However, the German Sepsis Society retained the SIRS criteria in their revision of the national sepsis guideline published in 2010 [12]. To the best of our knowledge, prospective data on sepsis recognition by ED physicians have not been published. Therefore, we conducted a prospective cohort study in our interdisciplinary ED, independently from ED physicians, and analyzed the following questions in retrospect: (1) How was sepsis recognized in the ED? (2) What are possible influencing factors on missed sepsis diagnoses? and (3) How do recognition and classification of sepsis affect quality of care, admission to the ICU, mortality, and length of hospital stay?

## Methods

### Study population and setting

**Fig. 1:**
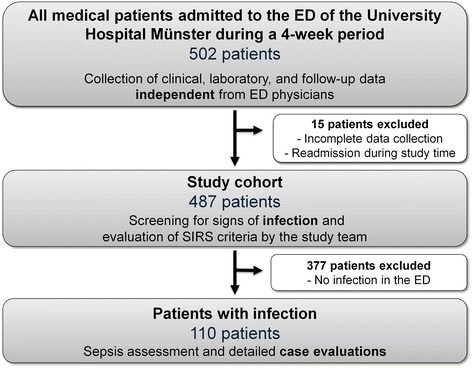
Study design. Generation of the study cohort and selection of patients with infection in the ED

In the multidisciplinary ED of the University Hospital Muenster, approximately 15,000 patients are treated per year, divided into about 6800 internal medical patients, 5200 neurological patients and 3000 patients of other divisions (e.g. urology or gynecology). Surgical, ophthalmologic or ear nose and throat (ENT) patients are treated in separate EDs. Our multidisciplinary ED is a Division of our Department of Medicine D, and thus managed by Internal Medicine. All patients are initially seen by an internal resident or attending physician according to the first-view-concept. All medical patients receive a detailed discharge letter upon transfer from the ED to the wards. A total of 502 consecutive internal medical patients ≥18 years of age presenting to the multidisciplinary ED of the University Hospital Muenster during a 4-week study period in 2013 were analyzed for this study. Fifteen patients were excluded because of incomplete data collection or readmission during study time (Fig. 1). The study was approved by the local ethics committee. Informed consent was waived because routine care was not influenced and no therapeutic intervention was performed.

### Study design

While some of the data was collected prospectively, this study was based on retrospective analysis of the electronic medical records and ED discharge letters to screen for cases of suspected infection (Fig. 1). These cases were then thoroughly reviewed by at least two independent internists (MM, AL, or PK) with regard to recognition and correct classification of sepsis (ACCP/SCCM definitions), adequate detection of the septic focus (history, examination, chest x-ray, urine dipstick, and eventually abdominal ultrasonography), empiric antibiotic regimen according to available national guidelines [13–16], appropriate fluid resuscitation and outcome (death, admission to an intensive care unit, length of hospital stay). In the event of disagreement, the case was discussed within the whole study team, and a final rating was set.

### Data collection

To simplify and guarantee full data collection, we implemented an additional study sheet that was filled out by the nursing staff without the involvement of ED physicians. Although the usual chart remained in the patient record, the study sheet was collected separately for the study team. It contained height, weight, complete vital signs, peripheral oxygen saturation, and oxygen supply. Vigilance was documented using the Alert-Voice-Pain-Unresponsive (AVPU) scale instead of the Glasgow Coma Scale for reasons of simplicity and because of its use in the Modified Early Warning Score (MEWS) [17]. The comparability of these two scales has already been analyzed [18, 19]. Patients were categorized by their best reaction to external stimuli (A for “Alert,” V for “Reacting to vocal stimuli,” P for “Reacting to pain stimuli,” U for “Unresponsive”).

To compare disease severity in different sepsis subgroups, we calculated the MEWS, consisting of several vital signs, and the AVPU score in all patients, as well as the modified Mortality in Emergency Department Sepsis (mMEDS) score [20, 21], which incorporates several risk factors (terminal illness, tachypnea/hypoxia, septic shock, platelet count <150,000/mm^**3**^, age >65 years, lower respiratory tract infection, nursing home residency, altered mental state). A contemporary version of the Charlson Comorbidity Index (CCI) was used to compare chronic disease burden [22].

Standardized blood measures included sodium, potassium, glucose, protein, bilirubin, aspartate aminotransferase, lactate dehydrogenase, creatine kinase, creatinine, C-reactive protein, blood count, coagulation status, and lactate levels. Laboratory analysis could be extended individually by the treating clinician.

### Sepsis severity definitions

Sepsis was defined according to the American College of Chest Physicians/Society of Critical Care Medicine (ACCP/SCCM) consensus criteria [6], which are still recommended for use in the current guidelines of the German Sepsis Society [12]:

- Sepsis was defined as SIRS (see introduction above) plus confirmed or suspected infection.
- Severe sepsis was defined as sepsis with at least one of the concomitant organ dysfunctions [23], as follows: *central nervous system* – new altered mental state and/or new onset AVPU worse than “Alert”; *respiratory* – supplemental oxygen required to maintain oxygen saturation >95% and respiratory rate >24/breaths per minute; *cardiovascular* – any vasopressor use required; *renal* – creatinine >50% of baseline or >2 mg/dL if baseline was unknown; *hepatic* – total serum bilirubin ≥4 mg/dL or alanine aminotransferase ≥2 times the upper limit of normal; *hematopoietic* – platelet count <100,000/μL; and *metabolic* – lactate >2.5 mmol/L.
- Septic shock was defined as sepsis with hypotension despite appropriate fluid resuscitation.

### Statistical analysis

Data are presented as absolute numbers, percentages, and medians with corresponding 25^th^ and 75^th^ percentiles (interquartile range; IQR). Differences between groups were analyzed by two-sided Mann–Whitney U tests or the Kruskal–Wallis test in case of continuous variables. Chi-square analysis was used to compare categorical variables. The distribution of the time-to-event variables was estimated using the Kaplan–Meier method with log-rank testing. Parameters associated with misclassification of sepsis were identified by univariate and multivariate binary logistic regression models. Variables found to be statistically significant at a 10% level in univariate analysis were included in the multivariate model. Two-sided *p*-values <0.05 were considered statistically significant. Data analysis was computed using SPSS (SPSS Inc, Chicago, IL, USA).

## Results

A total of 487 patients were eligible for sepsis screening by the study team (Fig. 1). The cohort showed a wide range of different leading symptoms and clinical pictures. Median age was 58 (IQR 41–72), and 197 (40.5%) of patients were women. Subsequent in-depth case evaluation revealed that 110 patients (22.6%) suffered from infection (Fig. 1). Additional file 1 shows baseline demographics and clinical variables in patients with and without infection (*n* = 377, 77.4%).

### Prevalence of sepsis in patients with infection

Patients with infection were subdivided into three categories according to disease severity: “non-SIRS” (defined as infection with <2 SIRS criteria), “sepsis,” and “severe sepsis” (Table 1). Of the 110 patients with infection, 54 patients (49%) fulfilled sepsis criteria, including 20 (18%) with organ dysfunction and thus severe sepsis. This post-hoc classification refers to the final assessment of the study team.

**Table 1:** Comparison of patients with infection regarding disease severity

Variable	Total	Non-SIRS	Sepsis	Severe sepsis	*P* value
Case characteristics					
Number of patients, *n* (%)	110 (100)	56 (50.9)	34 (30.9)	20 (18.2)	
Age in years, median (IQR)	59 (44–73)	61 (39–75)	57 (45–70)	62 (50–74)	0.5
Female sex, n (%)	46 (41.8)	25 (44.6)	14 (41.2)	7 (35.0)	0.8
C-reactive protein in mg/dL, median (IQR)	8.6 (2.8–14.6)	5.6 (1.7–12.3)	10.7 (4.5–15.3)	18.4 (7.4–26.4)	<0.0001
SIRS, mean/median (IQR)	1.6/1 (1–2)	0.6/1 (0–1)	2.5/2.5 (2–3)	2.7/2 (2–3)	<0.0001
MEWS, mean/median (IQR)	2.3/2 (1–4)	1.2/1 (0–2)	3.1 (1–4)	4.2/4 (2.5–6)	<0.0001
mMEDS, mean/median (IQR)	4.8/3 (2–8)	3.8/3 (0–5.5)	4.8/3 (2–9)	7.7/8 (4–11)	0.002
Focus of infection, *n* (%)					<0.0001
Respiratory tract	27 (24.5)	11 (19.6)	8 (23.5)	8 (40)	
Gastrointestinal tract	18 (16.4)	12 (21.4)	5 (14.7)	1 (5)	
Urinary tract	18 (16.4)	6 (10.7)	7 (20.6)	5 (25)	
Skin/soft tissue	8 (7.3)	5 (8.9)	2 (5.9)	1 (5)	
Other	6 (5.5)	5 (8.9)	0 (0)	1 (5)	
Focus of infection	33 (30)	17 (30.4)	12 (35.3)	4 (20)	
Organ dysfunctions, *n* (%)					
Present organ dysfunction	32 (29.1)	12 (21.4)	0 (0)	20 (100)	<0.0001
More than one organ dysfunction	8 (7.3)	1 (1.8)	0 (0)	7 (35)	

Median and interquartile range reported for continuous variables and frequency; percentage reported for categorical variables. Differences between groups (non-SIRS vs. sepsis vs. severe sepsis) were analyzed by two-sided Kruskal-Wallis test or chi-square test as appropriate. Two-sided *p*-values <0.05 were considered statistically significant. *BMI* body mass index, *MAP* mean arterial pressure, *SIRS* systemic inflammatory response syndrome, *MEWS* Modified Early Warning Score, *mMEDS* modified Mortality in Emergency Department Sepsis Score, *CCI* Charlson Comorbidity Index (2010 version)

Epidemiologic data and comorbid conditions were not different among the three groups. In contrast, C-reactive protein values, MEWS, and mMEDS steadily increased with severity across subgroups. Pulmonary infection (24.5%) was the most common source of infection and respiratory dysfunction (55%) was the predominant organ dysfunction in patients with severe sepsis (Table 1).

### Quality of care

Since the complete diagnostic workup and initiation of therapy is usually performed completely in our ED and not after admission to the ward, the following aspects regarding initial quality of care were evaluated:

#### Sepsis recognition and classification

**Fig. 2:**
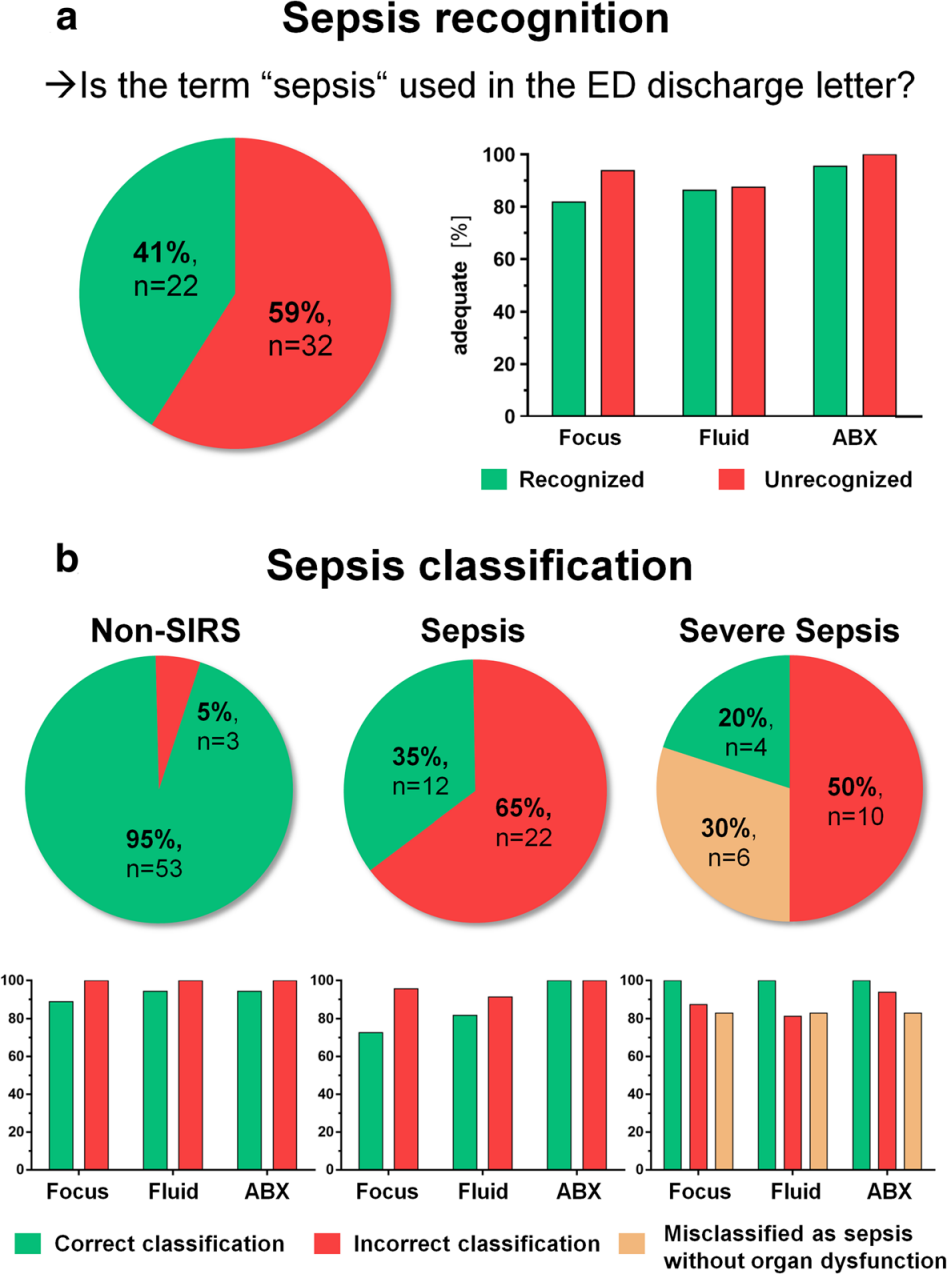
Quality of care according to sepsis recognition and classification. **a** Pie chart showing the percentage of recognized and unrecognized cases among all patients formally meeting SCCM/ACCP sepsis criteria (*n* = 54). Bar charts representing the proportion of adequate care as rated by the study team in retrospective case analysis. Focus: Detection of septic focus; Fluid: Fluid administration; ABX: Antibiotic regimen. **b** Pie charts showing the percentage of correctly classified disease severity within patients with suspected infection (*n* = 110), divided into non-SIRS (*n* = 56), sepsis (*n* = 34), and severe sepsis (*n* = 20). Bar charts depicting quality of care as described above

At first, we analyzed to what extent sepsis *recognition* by ED doctors (as documented in ED discharge letters) matched post-hoc classification by the study team. Given that organ dysfunction criteria are not standardized for the ED, we first analyzed if the term “sepsis” – in the broader sense – was used correctly in the subset of patients with sepsis or severe sepsis (Fig. 2a). The term was used correctly in only 22 of 54 patients (41%).

Sepsis was *classified* correctly (i.e., [non-SIRS] infection vs. sepsis vs. severe sepsis) in 69 of 110 patients (63%) from the infection cohort. However, correct classification declined across subgroups from 95% in non-SIRS infection to 35% and 20% in sepsis and severe sepsis, respectively (Fig. 2b).

Regarding the sepsis group, 12 of 34 cases (35%) were identified appropriately, while 22 sepsis diagnoses (65%) were overlooked and only revealed by the study team (Fig. 2b). Three patients were classified as septic in the absence of ≥2 positive SIRS criteria.

In the severe sepsis group, only 4 of 20 (20%) formally severe sepsis patients were denominated correctly, but another 6 patients (30%) were at least referred to as “sepsis,” missing only the organ dysfunction (Fig. 2b). One patient classified as having severe sepsis had preexisting thrombocytopenia and therefore did not meet the organ dysfunction criteria.

Multivariate binary logistic regression analysis revealed that the number of unlisted (albeit documented by the nurses) vital signs in the ED discharge letter [odds ratio (OR): 6.5, 95% confidence interval (CI): 1.2–34.1, *p* = 0.027; Additional file 2], in addition to the ability to stand [OR: 30.2 (95% CI: 2.8–323), *p* = 0.005] and higher systolic blood pressure [OR: 1.6, CI: 1.1–2.2, *p* = 0.01) were independently associated with a missed sepsis diagnosis (see table in Additional file 3). Interestingly, median disease severity (mMEDS 4 [IQR 2–9.5] vs. 6 [3–9], *p* = 0.455) and presence of terminal illness (25 vs. 29%, *p* = 0.773) were not different between unrecognized and recognized sepsis patients.

#### Detection of septic focus

The detection of the septic focus (history, examination, x-ray, ultrasonography, urine dipstick) to confirm or rule out a specific source of infection in the ED was considered to be adequate in 97 of 110 (88%) of patients with infection. However, the proportion of cases with adequate workup was not different between patients with recognized (18/22, 82%) or unrecognized (30/32, 94%) sepsis (*p* = 0.170) (Fig. 2a).

Additionally, collection of microbiological cultures (not rated) was not significantly different between patients with recognized (18/22, 82%) and unrecognized sepsis (20/32, 63%) (*p* = 0.127).

#### Therapy

Antibiotics were already started in the ED in 42 of 56 (75%) patients with non-SIRS, 30 of 34 (88%) patients with sepsis, and 19 of 20 (95%) patients with severe sepsis. The median duration from admission to administration of antibiotics tended to be shorter with increasing disease severity (non-SIRS: 3 h; sepsis: 2 h; severe sepsis: 1.5 h; *p* = 0.07), but was not statistically different between cases of recognized and unrecognized sepsis (1.5 h vs. 2 h, *p* = 0.985). The empiric antibiotic regimen was considered adequate in 53 of 56 patients without SIRS (95%), 33 of 34 patients with sepsis (97%), and 19 of 20 patients with severe sepsis (95%). The quality of antibiotic therapy did not differ statistically among the three categories of disease severity (*p* = 0.41).

Regarding sepsis recognition, the antibiotic strategy was rated appropriate in 21 of 22 of detected sepsis cases (96%) and all 32 cases of undetected sepsis (*p* = 0.42) (Fig. 2a).

Fluid administration was considered adequate in 51 of 56 patients without SIRS (91%), 30 of 34 patients with sepsis (88%), and 17 of 20 patients with severe sepsis (85%, *p* = 0.38), as well as in 19 of 22 (86%) recognized vs. 28 of 32 (88%) unrecognized sepsis cases (*p* = 0.38) (Fig. 2a). Furthermore, no significant differences in quality of care were evident regarding correctness of exact sepsis classification (Fig. 2b).

### Outcome

Hospital mortality was 5.5% (6 of 110) in patients with infection. Two of these six patients died in the ICU. In total, 6 patients were admitted to the ICU during the clinical course (1 with infection, 2 with sepsis, 3 with severe sepsis in the ED). The combined endpoint of death or admission to ICU was not different between cases of recognized (18%) and unrecognized sepsis (13%, *p* = 0.34).

**Fig. 3:**
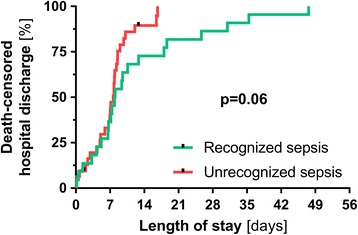
Death-censored length of hospital stay according to sepsis recognition. Kaplan–Meier curves with log-rank testing showing the length of stay in recognized (*n* = 22) and unrecognized (*n* = 32) patients with sepsis (ACCP/SCCM definitions)

Median length of stay was 7.1 (0.5–14.2) days in survivors with non-SIRS, 7.1 (4.4–8.5) days in survivors with sepsis, and 9.2 (7.1–14.7) days in survivors with severe sepsis. As shown by Kaplan–Meier curves with log-rank testing (Fig. 3), recognition of sepsis was not associated with median death-censored length of stay (8.0 vs. 7.6 days, *p* = 0.06). As a matter of fact, unrecognized sepsis cases even trended towards earlier hospital discharges. As shown by Additional file 4, this finding was also true for the subgroup of patients with severe sepsis.

## Discussion

This study clearly shows the existence of an appreciable gap illustrated by the high number of cases that formally meet the ACCP/SCCM sepsis criteria but are not well recognized by ED physicians. Only 41% of formal sepsis diagnoses were explicitly articulated in the patient record. At first glance, this result is alarming and definitely deserves further attention. Inadequate perception of (available) vital signs might be a causative factor in this phenomenon. Nevertheless, under-recognition and misclassification of sepsis were not associated with inferior quality of patient care and poor outcome.

Previous ED studies [24, 25] focused on the improvement of sepsis screening after staff training, with the consequence that physicians probably paid more attention to sepsis definition criteria. Furthermore, some retrospective sepsis studies used sepsis-related ICD coding [26] or the existence of blood cultures to identify cases in retrospect [27, 28]. Of those, only Stoneking et al. evaluated if sepsis was explicitly articulated in the patient record or if a sepsis order set was launched [28]. They found that patients with recognized sepsis received antibiotics sooner and more often than unrecognized cases of sepsis. However, the value of this result is limited by the fact that patients with false-negative blood cultures, patients without initial suspicion of infection, and patients in whom blood cultures were simply forgotten were disregarded, not to mention that evidence of bacteremia is not a prerequisite for diagnosing sepsis. To avoid any potential selection bias, all medical patients presenting to our ED during a 4-week period for suspicion of infection were screened. Moreover, ED physicians were not aware of the study purpose so that study-triggered sepsis awareness could be avoided. To the best of our knowledge, such unique data on sepsis recognition by ED physicians have not been published previously.

We were surprised to see that non-recognition of formal ACCP/SCCM definitions had no deleterious impact on quality of care and short-term outcome in our study population. In their retrospective study, Stoneking et al. [28] found a significantly higher proportion of initiated antibiotic therapy (91.9 vs. 75.8%, overall 85.0%, *p* = 0.002), lower median time to antibiotics (2.4 h vs 3.81 h, *p* = 0.002), and a trend towards higher total volume of fluid resuscitation in patients with recognized (i.e., articulated) sepsis. However, outcome was not different between recognized and unrecognized sepsis, respectively. Patients in our cohort generally received antibiotics more often (overall: 90.7%) and earlier (overall: median 2.5 h). This quick administration of antibiotics, together with rigorous diagnostics to detect the septic focus and guideline-conforming therapy in both recognized and unrecognized sepsis, might explain why outcomes did not differ between the groups.

Incomplete listing of vital signs in the ED discharge letter could be identified as an independent risk factor for missing sepsis diagnoses. Given that ACCP/SCCM sepsis definitions are fundamentally based on altered vital signs, incomplete documentation suggests a lack of appreciation or maybe even ignorance of sepsis definitions. In a retrospective study from the Netherlands [26] fragmentary collection of vital signs (only 7.6% were documented completely) was associated with rare sepsis recognition (14% of cases). Even though acute patient care is more important than paperwork, poor record keeping may promote underdiagnosis of sepsis.

After completion of this study, new consensus definitions of sepsis (Sepsis-3) were published in 2016 by the ESICM-SCCM Sepsis Redefinitions Task Force [29]. Sepsis is now defined as a “life-threatening organ dysfunction due to a dysregulated host response to infection.” In this new definition, the concept of the non-homeostatic host response to infection is strongly stressed while the SIRS criteria have been removed and ‘severe sepsis’ no longer exists. Now there is simply ‘sepsis’ (quite similar to the concept of severe sepsis) and ‘septic shock,’ whereas the former (non-severe) ‘sepsis’ is no longer included in the new classification. Our results suggest that the latter can be safely omitted in ED patients. We were also curious if Sepsis-3 definitions match actual sepsis recognition by ED doctors better than old sepsis criteria. Preliminary analysis revealed that 22 patients with non-severe sepsis were dropped out while 17 patients (all SIRS-negative but with organ damage) had sepsis according to Sepsis-3 definitions. However, sepsis was articulated in only 35% of Sepsis-3–positive cases, and recognition was thus even worse than with the old definitions (41%). Nevertheless, death censored length of hospital stay still did not differ between recognized and unrecognized sepsis (Kaplan-Meier curves are shown in Additional file 5).

Several limitations deserve discussion. First of all, our study was a rather small single-center study, but the immense effort of prospectively assessing all included patients and especially the detailed case evaluations precluded a considerably larger sample size. Although the different subgroups had only a small number of patients, we found consistent and homogeneous results. However, our findings cannot be readily extrapolated to the patient populations of other EDs. Second, although results from our case evaluations (two-person rule) were highly concordant, some variables (e.g., appropriateness of fluid therapy) are difficult to gauge in retrospect. Third, information about physician recognition was derived only from clinical reports. It therefore remains unclear whether “sepsis” was simply not articulated in the reports but was the working assumption in the ED. Because the case evaluations were performed in retrospect, one cannot differentiate between misrecognition and misdocumentation. This issue can hardly be overcome, because any prospective evaluation (e.g. real-time observation and/or interviews) would potentially confound the doctor’s performance in the ER (hawthorne effect). Fourth, because of low event rates in this study, the outcome criteria death and admission to the intensive care unit must be interpreted very carefully. However, the overall in-hospital mortality (7.4%) in patients with sepsis and severe sepsis was comparable to previous prospective ED studies (Gille-Johnson [27]: 2.7%; Shapiro [30]: 1.3%/9.2%). Finally, the length of hospital stay is probably influenced by various factors. Nevertheless, it is conceivable that undertreatment of unrecognized sepsis patients leads to an increased length of hospital stay (which was not the case in this study).

## Conclusions

Our results suggest that a lot of ED patients with serious infections who formally meet organizational sepsis definitions are not recognized or documented as septic in the ED. Nevertheless, non-recognition of sepsis seemed to have no deleterious impact on initial therapy adequacy in the ED. So is recognition and/or correct classification of sepsis in the ED dispensable after early antibiotics and crystalloid resuscitation, as recommended by the Surviving Sepsis Campaign, have become usual care in all patients with infection in the ED? Although our data cannot fully answer this question, we strongly believe, that complete documentation and review of vital signs is of utmost importance in the ED. This is especially important for the proposed use of the quick Sepsis-Related Organ Failure Assessment score (known as qSOFA) [29], to screen for ED patients with suspected infection who are at greater risk for a poor outcome outside the ICU.

## Additional files

Additional file 1: Baseline characteristics of all included patients (table). (PDF 169 kb)

Additional file 2: Percentage of documented vital signs in the ED discharge letter for recognized (*n* = 22) and unrecognized (*n* = 32) cases of sepsis. BP, blood pressure; HR, heart rate; RR, respiratory rate; T, temperature; S_p_O_2_, peripheral oxygen saturation. (PDF 221 kb)

Additional file 3: Univariate and multivariate regression analysis for impact on missing sepsis diagnoses (table). (PDF 88 kb)

Additional file 4: Death-censored length of hospital stay according to severe sepsis recognition (ACCP/SCCM definitions). Kaplan-Meier curves with Log-rank testing showing the length of stay in recognized (*n* = 10) and unrecognized (*n* = 10) patients with severe sepsis. (PDF 115 kb)

Additional file 5: Death-censored length of hospital stay according to sepsis recognition (Sepsis-3 definitions). Kaplan-Meier curves with log-rank testing showing the length of stay in recognized (*n* = 17) and unrecognized (*n* = 32) patients with sepsis. (PDF 117 kb)

## Abbreviations

AVPU-Scale: Alert-Voice-Pain-Unresponsive scale
CCI: Charlson comorbidity index
ED: Emergency department
ENT: Ears, nose and throat
ICD: International classification of diseases
ICU: Intensive care unit
IQR: Interquartile range
MEWS: Modified early warning score
mMEDS: Modified mortality emergency department sepsis score
OR: Odds ratio
qSOFA-Score: Quick sepsis-related organ failure assessment score
SIRS: Systemic inflammatory response syndrome
