# Supplementary material for: Sepsis recognition in the emergency department – impact on quality of care and outcome?
Source: BMC Emerg Med. 2017 Mar 23;17:11. doi: 10.1186/s12873-017-0122-9 (PMC5363055; doi:10.1186/s12873-017-0122-9)
Supplement: Supplementary file 1 — Baseline characteristics of all included patients (table). (PDF 169 kb) [file 12873_2017_122_MOESM1_ESM.pdf]

Additional file 1

Sepsis recognition in the emergency department - impact on quality of care and outcome?

Marius Morr, Alexander Lukasz, Eva Rübig, Hermann Pavenstädt, Philipp Kümpers

Baseline characteristics of all included patients

Variable	Total	No infection	Infection	P value
**Demographics**				
Number of patients, n (%)	487 (100)	377 (77.4)	110 (22.6)	
Age in years, median (IQR)	58 (41–72)	57 (40–72)	59 (44–73)	0.5
Female sex, n (%)	197 (40.5)	151 (40.1)	46 (41.8)	0.7
**Primary reason for admission, n (%)**				
Cardiovascular		203 (53.8)	0	
Gastrointestinal		53 (14.1)	0	
Respiratory		37 (9.8)	0	
Nephrologic/ rheumatologic		31 (8.2)	0	
Hematologic		12 (3.2)	0	
Intoxication/drug-related		9 (2.4)	0	
Other		32 (8.5)	0	
Infection		0	110 (100)	
**SIRS criteria**				
Positive SIRS criteria, n (%)				<0.0001
0	264 (54.2)	244 (64.7)	20 (18.2)	
1	143 (29.4)	107 (28.4)	36 (32.7)	
2	49 (10.1)	21 (5.6)	28 (25.5)	
3	26 (5.3)	5 (1.3)	21 (19.1)	
4	5 (1)	0 (0)	5 (4.5)	
SIRS, mean/median (IQR)	0.7/0 (0–1)	0.4/0 (0–1)	1.6/1 (1–2)	<0.0001
Heart rate in beats per min, median (IQR)	80 (68–96)	77 (66–92)	92 (80–106)	<0.0001

Respiratory rate, breaths/min (IQR)	14 (12–18)	14 (12–17)	16 (13–20)	<0.0001
Temperature in °C, median (IQR)	36.2 (36.0–36.7)	36.2 (36.0–36.5)	37.2 (36.1–38.4)	<0.0001
Leukocytes (×1000/µL)	8.1 (6.2–10.3)	7.8 (6.2–9.7)	10 (5.8–13.7)	0.006
**Additional clinical data**				
MAP in mmHg, median (IQR)	98 (90–109)	101 (92–111)	92 (83–102)	<0.0001
CRP in mg/dL, median (IQR)	<0.5 (<0.5–3.5)	<0.5 (<0.5–0.9)	8.6 (2.8–14.6)	<0.0001
Lactate mmol/L	0.9 (0.7–1.5)	0.9 (0.7–1.5)	0.9 (0.7–1.4)	0.9
Hospitalization, n (%)	159 (53.2)	168 (44.6)	91 (82.7)	<0.0001

Median and interquartile range reported for continuous variables and frequency; percentage reported for categorical variables. Differences between patients with/without infection were calculated using the Mann–Whitney U test for continuous variables and chi-square test for categorical variables. Two-sided p values <0.05 were considered statistically significant. SIRS, systemic inflammatory response syndrome; MAP, mean arterial pressure; CRP, C-reactive protein.
