# Supplementary material for: Sepsis recognition in the emergency department – impact on quality of care and outcome?
Source: BMC Emerg Med. 2017 Mar 23;17:11. doi: 10.1186/s12873-017-0122-9 (PMC5363055; doi:10.1186/s12873-017-0122-9)
Supplement: Supplementary file 2 — Percentage of documented vital signs in the ED discharge letter for recognized (n = 22) and unrecognized (n = 32) cases of sepsis. BP, blood pressure; HR, heart rate; RR, respiratory rate; T, temperature; SpO2, peripheral oxygen saturation. (PDF 221 kb) [file 12873_2017_122_MOESM2_ESM.pdf]

Additional file 2

Sepsis recognition in the emergency department - impact on quality of care and outcome?

Marius Morr, Alexander Lukasz, Eva Rübig, Hermann Pavenstädt, Philipp Kümpers

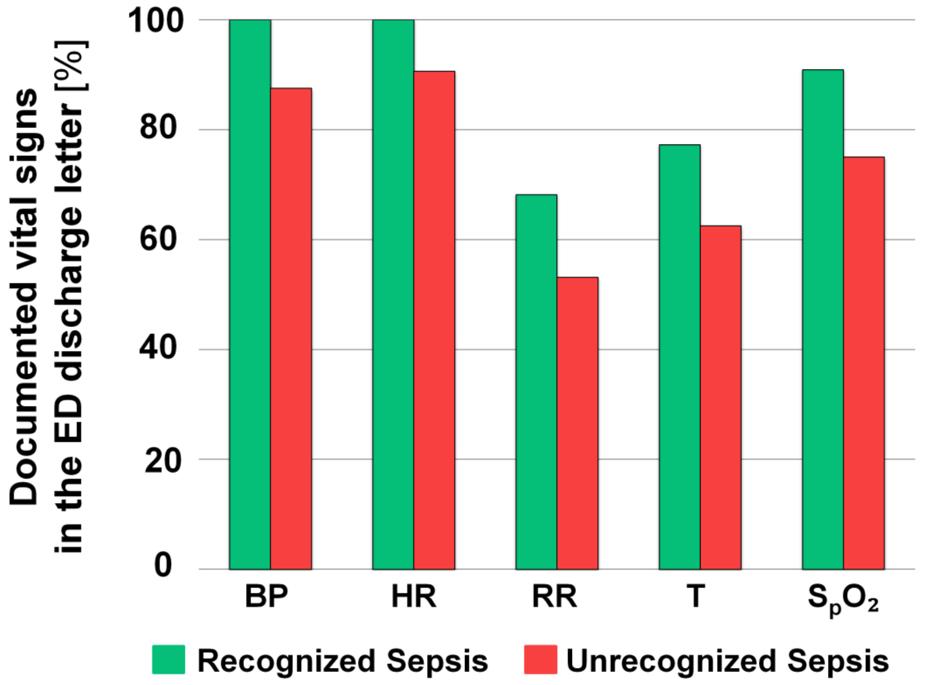

Percentage of documented vital signs in the ED discharge letter for recognized (n=22) and unrecognized (n=32) cases of sepsis. BP, blood pressure; HR, heart rate; RR, respiratory rate; T, temperature; S_pO_2, peripheral oxygen saturation.
