# Supplementary material for: Sepsis recognition in the emergency department – impact on quality of care and outcome?
Source: BMC Emerg Med. 2017 Mar 23;17:11. doi: 10.1186/s12873-017-0122-9 (PMC5363055; doi:10.1186/s12873-017-0122-9)
Supplement: Supplementary file 3 — Univariate and multivariate regression analysis for impact on missing sepsis diagnoses (table). (PDF 88 kb) [file 12873_2017_122_MOESM3_ESM.pdf]

Additional file 3

Sepsis recognition in the emergency department - impact on quality of care and outcome?

Marius Morr, Alexander Lukasz, Eva Rübig, Hermann Pavenstädt, Philipp Kümpers

Univariate and multivariate regression analysis for impact on missing sepsis diagnoses

Variable	Univariate		Multivariate	
-	OR (95% CI)	P-value	OR (95% CI)	P-value
Systolic blood pressure (per 10mmHg)	1.45 (1.09-1.93)	0.012	1.62 (1.14-2.30)	0.007
Ability to stand	5.94 (1.65-21.41)	0.006	18.46 (2.67-127.81)	0.003
Number of undocumented vital signs	1.66 (0.95-2.91)	0.075	3.88 (1.22-12.30)	0.021
MEWS	0.72 (0.54-0.97)	0.028	0.73 (0.47-1.14)	0.337
MAP	1.05 (1.01-1.10)	0.045	0.928 (0.82-1.05)	0.226

Odds ratios (OR), 95% confidence intervals (CI), and p-values were calculated using logistic regression analysis (backward wald method). Variables were selected a priori based on theoretical considerations and existing literature. Those statistically significant at the 10% level in the univariate analysis were included in the multivariate model. Two-sided p values < 0.05 were considered statistically significant in the multivariate model. MEWS, Modified Early Warning Score. MAP, Mean Arterial Pressure.
