# Supplementary material for: Sepsis recognition in the emergency department – impact on quality of care and outcome?
Source: BMC Emerg Med. 2017 Mar 23;17:11. doi: 10.1186/s12873-017-0122-9 (PMC5363055; doi:10.1186/s12873-017-0122-9)
Supplement: Supplementary file 4 — Death-censored length of hospital stay according to severe sepsis recognition (ACCP/SCCM definitions). Kaplan-Meier curves with Log-rank testing showing the length of stay in recognized (n = 10) and unrecognized (n = 10) patients with severe sepsis. (PDF 115 kb) [file 12873_2017_122_MOESM4_ESM.pdf]

Additional file 4

Sepsis recognition in the emergency department - impact on quality of care and outcome?

Marius Morr, Alexander Lukasz, Eva Rübig, Hermann Pavenstädt, Philipp Kümpers

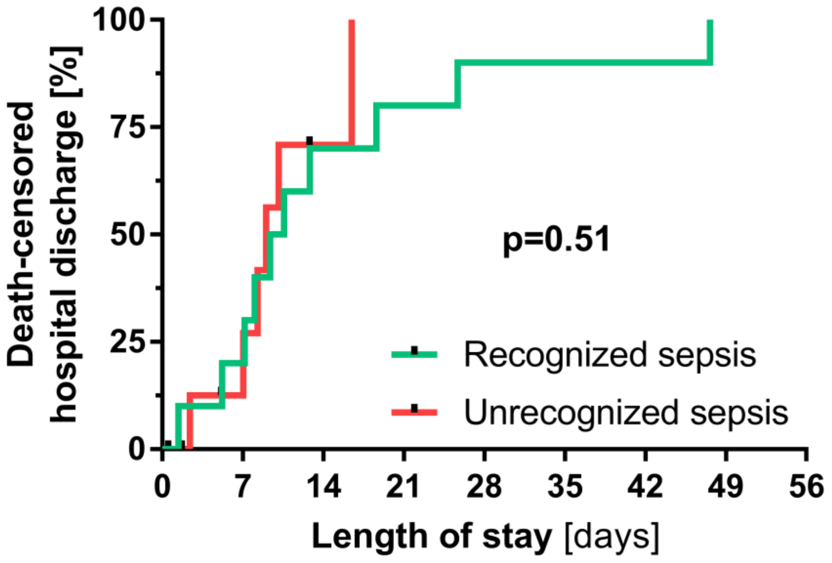

Death-censored length of hospital stay according to severe sepsis recognition (ACCP/SCCM definitions). Kaplan-Meier curves with Log-rank testing showing the length of stay in recognized (n=10) and unrecognized (n=10) patients with severe sepsis.
