# Supplementary material for: Sepsis recognition in the emergency department – impact on quality of care and outcome?
Source: BMC Emerg Med. 2017 Mar 23;17:11. doi: 10.1186/s12873-017-0122-9 (PMC5363055; doi:10.1186/s12873-017-0122-9)
Supplement: Supplementary file 5 — Death-censored length of hospital stay according to sepsis recognition (Sepsis-3 definitions). Kaplan-Meier curves with log-rank testing showing the length of stay in recognized (n = 17) and unrecognized (n = 32) patients with sepsis. (PDF 117 kb) [file 12873_2017_122_MOESM5_ESM.pdf]

Additional file 5

Sepsis recognition in the emergency department - impact on quality of care and outcome?

Marius Morr, Alexander Lukasz, Eva Rübig, Hermann Pavenstädt, Philipp Kümpers

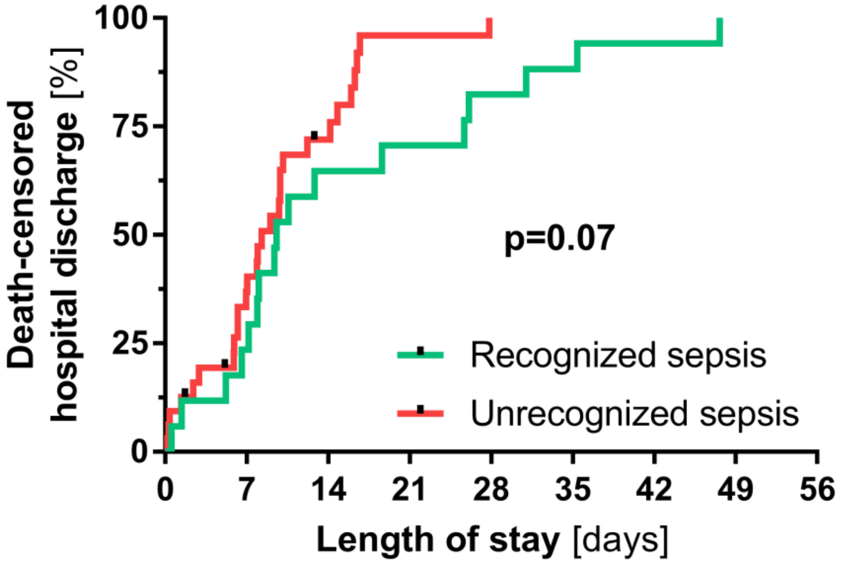

Death-censored length of hospital stay according to sepsis recognition (Sepsis-3 definitions). Kaplan-Meier curves with log-rank testing showing the length of stay in recognized (n=17) and unrecognized (n=32) patients with sepsis.
